# Increased gut permeability and intestinal inflammation precede arthritis onset in the adjuvant-induced model of arthritis

**DOI:** 10.1186/s13075-023-03069-9

**Published:** 2023-06-06

**Authors:** Sophie Hecquet, Perle Totoson, Hélène Martin, Marie-Paule Algros, Philippe Saas, Jean-Paul Pais-de-Barros, Alban Atchon, Benoît Valot, Didier Hocquet, Maude Tournier, Clément Prati, Daniel Wendling, Céline Demougeot, Frank Verhoeven

**Affiliations:** 1 Université de Franche-Comté, PEPITE, 25000 Besançon, France; 2 Service de Rhumatologie, CHRU Besançon, 25000 Besançon, France; 3 Service d’anatomopathologie, CHRU Besançon, 25000 Besançon, France; 4 UMR1098 RIGHT, Université de Franche-Comté, INSERM, EFS BFC, 25000 Besançon, France; 5 Lipidomic Analytic Platform LabEX LipSTIC, INSERM, LNC UMR1231, F-21000 Dijon, France; 6 Université de Bourgogne, INSERM UMR1231, 21000 Dijon, France; 7 Université de Franche-Comté, Bioinformatique Et Big Data Au Service de La Santé, UFR Sciences de La Santé, 25000 Besançon, France; 8 Université de Franche-Comté, UMR CNRS 6249 Chrono-Environnement, 25030 Besançon, France; 9 Université de Franche-Comté, EPILAB EA, 4266 Pathogènes Et Inflammation, 25000 Besançon, France

**Keywords:** Intestinal permeability, Intestinal inflammation, Dysbiosis, Microbiota, Animal models

## Abstract

**Background:**

Intestinal inflammation, dysbiosis, intestinal permeability (IP), and bacterial translocation (BT) have been identified in patients with spondyloarthritis but the time at which they appear and their contribution to the pathogenesis of the disease is still a matter of debate.

**Objectives:**

To study the time-course of intestinal inflammation (I-Inf), IP, microbiota modification BT in a rat model of reactive arthritis, the adjuvant-induced arthritis model (AIA).

**Methods:**

Analysis was performed at 3 phases of arthritis in control and AIA rats: preclinical phase (day 4), onset phase (day 11), and acute phase (day 28). IP was assessed by measuring levels of zonulin and ileal mRNA expression of zonulin. I-inf was assessed by lymphocyte count from rat ileum and by measuring ileal mRNA expression of proinflammatory cytokines. The integrity of the intestinal barrier was evaluated by levels of iFABP. BT and gut microbiota were assessed by LPS, soluble CD14 levels, and 16S RNA sequencing in mesenteric lymph node and by 16S rRNA sequencing in stool, respectively.

**Results:**

Plasma zonulin levels increased at the preclinical and onset phase in the AIA group. Plasma levels of iFABP were increased in AIA rats at all stages of the arthritis course. The preclinical phase was characterized by a transient dysbiosis and increased mRNA ileal expression of IL-8, IL-33, and IL-17. At the onset phase, TNF-α, IL-23p19, and IL-8 mRNA expression were increased. No changes in cytokines mRNA expression were observed at the acute phase. Increased CD4^+^ and CD8^+^ T cell number was measured in the AIA ileum at day 4 and day 11. No increase in BT was observed.

**Conclusion:**

These data show that intestinal changes precede the development of arthritis but argue against a strict “correlative” model in which arthritis and gut changes are inseparable.

**Supplementary Information:**

The online version contains supplementary material available at 10.1186/s13075-023-03069-9.

## Key messages

• Intestinal inflammation and alterations of the intestinal barrier are not the consequence of joint damage

• Intestinal changes occurred in the absence of bacterial translocation

**Supplementary Information:**

The online version contains supplementary material available at 10.1186/s13075-023-03069-9.

## Introduction

Growing evidence argues for the role of the gut in the pathophysiology of spondyloarthritis (SpA). Recent data from clinical and basic research suggested that the microbiota and the integrity of the intestinal barrier might be key elements in the disruption of immune tolerance. This so-called gut-joint axis involves dysbiosis and bacterial translocation, intestinal inflammation, and increase in intestinal permeability [1, 2]. While abundant literature is available on dysbiosis, less is known on hyperpermeability. Thus, an increase in intestinal permeability has been described in SpA patients, but the majority of available studies are dated and have a small sample size [3]. Recent data on this subject showed that SpA patients had an increase in serum zonulin levels, a precursor of haptoglobin 2 and a physiological modulator of intestinal epithelial tight junctions [2]. Even though the presence of this inseparable tryptic (dysbiosis/intestinal hyperpermeability/gut inflammation) is undisputable in SpA, the exact link between these elements is unclear. The first hypothesis is that of a “causal” relationship. According to this hypothesis, dysbiosis would induce increased intestinal permeability and gut inflammation, leading to the activation of the immune system and its spreading to the joint [4]. However, data did not support this hypothesis. Indeed, anti-integrin therapy used in inflammatory bowel diseases led to the onset or flares of SpA, and therapies targeting IL-17A were effective in the joint but not in the gut [5–7]. The second hypothesis is the “correlative” one meaning that arthritis and gut changes co-exist in patients in which immunological mechanisms are induced in the gut and joint. Adding to the complexity, whether or not a causal relationship exists between dysbiosis, gut inflammation and alterations in gut barrier function is still unknown [8].

Exploring the complex links between the gut and SpA in clinical studies is challenging, and this research could benefit from animal models that allow the study of the digestive tract in drug-free animals at different stages of disease progression, including pre-clinical stages. To date, no animal studies investigated together the link between arthritis, intestinal permeability, intestinal inflammation, and bacterial translocation. Studies on the HLA-B27 transgenic rats and SKG models of SpA showed that dysbiosis preceded the onset of arthritis, but no study was able to confirm this observation in humans [9, 10].

Of interest, recent data from rats with adjuvant-induced arthritis (AIA) identified the presence of endothelial dysfunction in mesenteric arteries at the onset of arthritis, suggesting the existence of early intestinal involvement in this model [11]. In this model, arthritis is mediated by an antigen, *Mycobacterium butyricum*. Indeed, it has been shown that in Lewis rats, T cells activated by Mycobacterium proliferate in co-culture with or without an antigen-presenting cell in contrast to T cells from non-immunized rats [12]. In AIA rats, the induction of arthritis by bacterial antigens suggests that this model is a model of reactive arthritis-type spondyloarthritis. To date, there are no studies evaluating intestinal manifestations in this model.

Thus, the aim of the present study was to investigate the time course of intestinal barrier integrity and permeability, intestinal inflammation, dysbiosis, and bacterial translocation at different stages of arthritis in the AIA model (preclinical, onset, and acute phase).

## Methods

For more details, see supplemental methods.

### Animal model

Six-week-old male Lewis rats (*n* = 150) were purchased from Janvier (Le Genest Saint Isle, France). Animals were kept on a 12-h light/dark cycle and allowed free access to food and water. The study was approved by the local committee for ethics in animal experimentation no. 2019–003-PT-5 PR of Franche-Comté University (Besançon, France), was conformed with the Animal Research: Reporting of In Vivo Experiments (ARRIVE) guidelines and complied with the Guide for the Care and Use of Laboratory Animals published by the US National Institutes of Health (publication No. 85–23, revised 2011).

### Induction, clinical follow-up, and evaluation of arthritis

Arthritis was induced in rats by a single intradermal injection at the base of the tail of 120 μl of 1 mg of heat-killed *Mycobacterium butyricum* suspended in 0.1 ml of Freund’s incomplete adjuvant. Non-arthritic rats were used as controls and received the same volume of saline. Rats were weighed and examined 5 days per week to assess an arthritis score (maximum arthritis score of 6 for each rat) [13].

### Experimental groups

Experiments were conducted on two series of rats: one was used for the study of intestinal and mesenteric lymph node microbiota (*n* = 60, 10 rats for each time studied, in the AIA group and in the control group), the other was used for analyses of serum, plasma, and ileal parameters (*n* = 90, 15 rats for each time studied, in the AIA group and in the control group). Groups were sacrificed at different times from *M. butyricum* or saline inoculation, corresponding to different stages of the disease [14]: Day 4 (preclinical group) represents the preclinical expression of arthritis and a time point during antigen processing, day 11 (onset group) represents the clinical onset of arthritis (very early arthritis) and day 28 (acute group) corresponds to severe active disease with severe joint swelling and destruction. At each time (preclinical, onset, acute) in the development of arthritis, one control group and one AIA group were used.

### Tissue collection

Rats were anesthetized with sodium pentobarbital (60 mg/kg, i.p.) and blood was withdrawn from the abdominal artery to obtain plasma and serum, stored at − 80 °C until analysis. Hind paws were collected and preserved in a 4% formalin solution until radiological analysis. Ileums were collected, one part was fixed in a 4% formalin solution and embedded into paraffin for histological analysis, and another part was preserved for 24 h in RNA-later and then frozen at − 80 °C for real-time quantitative (RT-q)PCR. The feces of the rats, removed from the rectal ampulla, and the mesenteric lymph nodes (MLN) were collected at the time of sacrifice and then were immediately frozen at − 80 °C.

### Radiographic ex vivo analysis

Radiographs of the hind paws were obtained with the BMA High-Resolution Digital X-Ray (40 mV, 10 mA)—D3A Medical Systems (France). A score from 0 to 20 was assigned for each paw according to the grading scale modified from Ackerman et al. [15]. This score used the scale of 0 (normal), 1 (slight), 2 (mild), 3 (moderate), and 4 (severe) to describe modifications for each of the five characteristic features of AIA. Radiographs took into account soft tissue swelling, demineralization as measured by bone density, loss of cartilage shown by narrowing of the joint spaces, bone erosions, and bone formation defined as the proliferation of new bone tissue. The maximum score for each rat is 40 (Suppl. Figure 1).

### Blood analysis

Intestinal permeability and integrity of the intestinal barrier were evaluated by measuring plasma levels of zonulin and intestinal fatty acid binding protein (iFABP), respectively. Bacterial translocation was assessed by measuring plasma levels of LPS by liquid chromatography coupled mass spectrometry (LCMS2) and serum levels of soluble CD14 (sCD14) by ELISA.

### Immunohistology of ileum

Paraffin-embedded ileum was cut in 4-μm-thick sections, and cells expressing CD3, CD4, or CD8 markers were visualized using the Ventana Ultraview DAB Detection System. For quantification of immune cells, sections were digitally scanned (Nanozoomer 2.0, Hamamatsu) and imported into QuPath 0.2.3 software (Suppl. Figure 2).

### Quantitative real-time polymerase chain reaction

Total RNA was extracted using Qiagen® mRNA extraction kit and RT-qPCR were performed using Biorad® cDNA and SybrGreen kits. The sequences of the primers used to study the expression of the mRNA of zonulin, TLR-4, intestinal junction proteins (ZO-1, occludin), and cytokines/chimiokines (CXCL-1, IL-33, IL-17A, IL23p19, TNF-α) as well as β-actin and GAPDH used as references are referenced below (Supplementary table 1).

### Metagenomic analysis of microbiota

A sequencing of the V3-V4 region of 16S rRNA was performed on Illumina MiSeq (v2) in pair-end (2 × 250 bp) for 60 fecal and 60 MLN samples (10 per each group). The identified ASVs were assigned taxonomy via the IDTAXA method [16]. For the 20 samples at D4 (AIA and controls), a compositional analysis with the package "ALDEx2" was performed.

### Statistical analysis

Values are expressed as the mean ± SEM. GraphPad Prism version 5.0 software was used for statistical analysis. Comparison between values of AIA and control groups was assessed using unpaired Student’s *t* test or Mann–Whitney test when data were not normally distributed. A non-parametric multivariate analysis of variance (PERMANOVA) approach was used to compare dissimilarities between bacterial communities. A compositional analysis [17] of the gut microbiota was performed to distinguish differentially abundant taxa between AIAs and controls. The relationship between two quantitative variables was investigated using Spearman's correlation coefficient. *P* values less than 0.05 were considered significant.

## Results

### Clinical and radiographical characteristics

As compared to controls (Table 1), a decrease in body weight was observed in AIA rats from day 4 (preclinical) to the acute stage of arthritis. The first signs of arthritis appeared on day 11 post-induction. Then clinical inflammation worsened to reach a maximum at day 18 (not shown), with the arthritis score remaining high until day 28. Paw diameters had the same evolution as arthritis score. Radiographic score revealed the presence of slight but significative radiographic damages on day 11, becoming severe on day 28 (Table 1).

**Table 1:** Clinical and structural parameters

	**Before induction day**		**Post-induction day**	
	**D0**	**D4 (preclinical)**	**D11 (onset)**	**D28 (acute)**
**Body weight (g)**				
Controls	178 ± 7	208 ± 14	248 ± 3	309 ± 3
AIA	189 ± 4	192 ± 5*	216 ± 4***	207 ± 3***
**Paw diameter (mm)**				
Controls	6.8 ± 0.1	7.1 ± 0.1	7.2 ± 0.1	7.2 ± 0.1
AIA	6.3 ± 0.4	6.9 ± 0.1	7.9 ± 0.2**	9.4 ± 0.3***
**Arthritis score**				
Controls	0	0	0	0
AIA	0	0	1.9 ± 0.9	3.9 ± 1.0
**Radiographic score**				
Controls	0	0.3 ± 0.5	0.3 ± 0.5	0.07 ± 0,3
AIA	0	0.3 ± 0.5	4.1 ± 1.7***	23.8 ± 8.1***

Values expressed as means ± SEM (*N* = 15 rats per group). *n.d.* not determined

^*^(*p* < 0.05). **(*p* < 0.005) and ***(*p* < 0.0005) different from age-matched controls

### Intestinal inflammation preceded the onset of arthritis

**Fig. 1:**
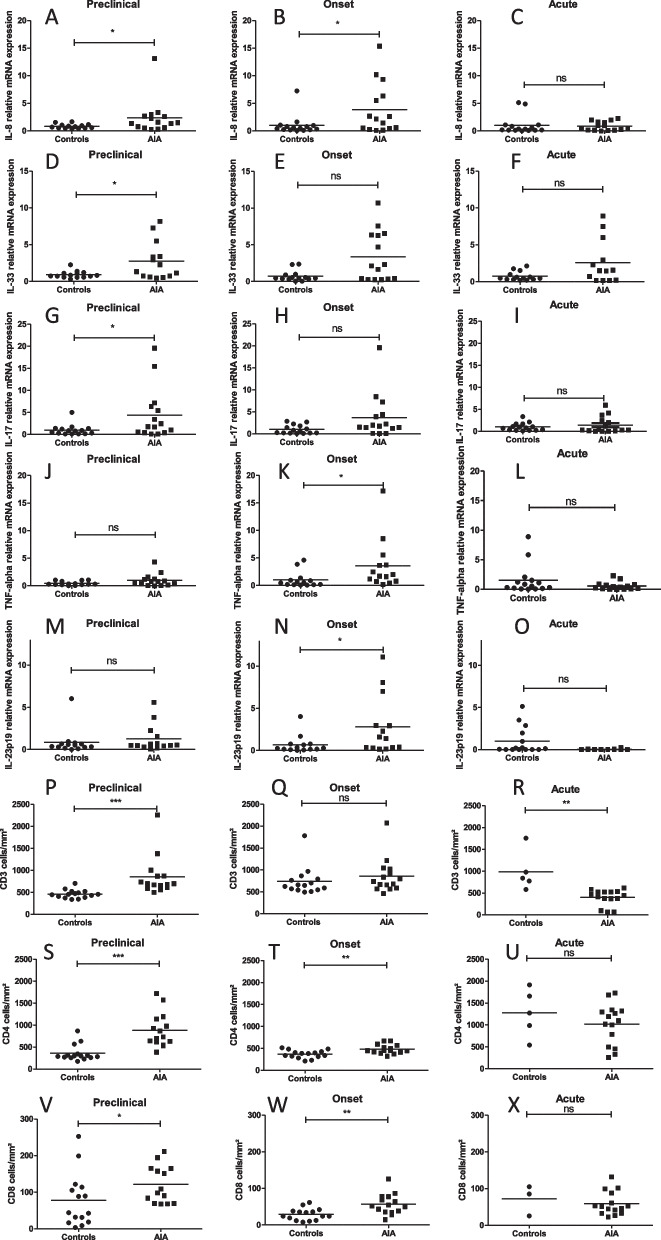
Intestinal inflammation in AIA rats. RT-qPCR analyses of pro-inflammatory cytokines mRNA expression in the ileum. At day 4 (preclinical), 11 (onset), and 28 (acute), analyses of IL-8 (**A**, **B**, **C**), IL-33 (**D**, **E**, **F**), IL-17A (**G**, **H**, **I**), TNF-α (**J**, **K**, **L**), and IL-23p19 (**M**, **N**, **O**) mRNA expression were performed (*n* = 14–15/group). At day 4 (preclinical), 11 (onset), and 28 (acute), immunohistochemical analyses of CD3 + (**P**, **Q**, **R**), CD4 + (**S**, **T**, **U**), and CD8 + (**V**, **W**, **X**) were performed (*n* = 10/group). The number of cells/mm^2^ was evaluated as described in supplementary methods. Results are expressed as means ± SEM. *(*p* < 0.05), **(*p* < 0.005). n.s., not significant

Consistent with a very early ileal inflammation, AIA rats exhibited significantly higher CXCL-1 (IL-8), IL-33, and IL-17A mRNA expression at the preclinical phase (Fig. 1A, D, G) compared to controls. At the onset of arthritis, IL-8, TNF-α, and IL-23p19 mRNA expression was increased in AIA rats (Fig. 1B, K, N). At the acute phase, no difference in cytokines mRNA expression was observed between AIA rats and controls (Fig. 1C, F, I, L, O). Immunohistochemical analysis showed that the ileal number of CD3^+^ T lymphocytes —including CD4^+^ and CD8^+^ cells — was increased at the preclinical phase in AIA rats as compared to controls (Fig. 1P, S, V). Similar results were obtained at the onset of arthritis (Fig. 1T, W) except for CD3^+^ T cells that were no longer different from controls (Fig. 1Q). At the acute phase, the number of CD3^+^ T cells was lower in AIA rats than in controls (Fig. 1R) whereas CD4^+^ and CD8^+^ cells were not different between groups (Fig. 1U, X).

### Increased intestinal permeability preceded the onset of arthritis

**Fig. 2:**
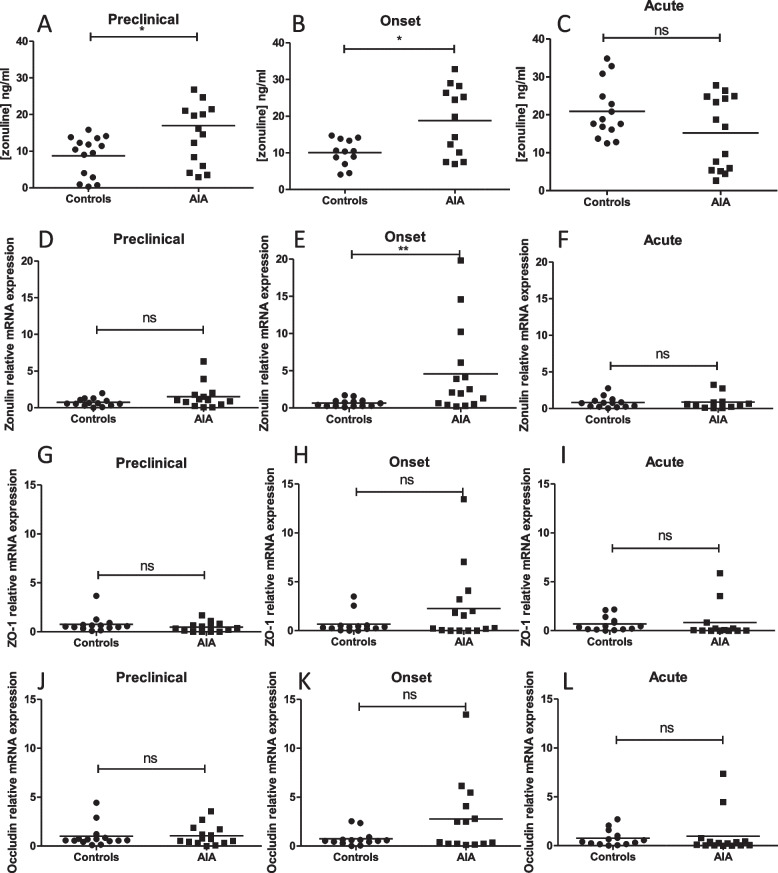
Intestinal permeability in AIA rats. Plasma zonulin levels were measured in controls and AIA rats at the preclinical (**A**), onset (**B**), and acute (**C**) stages. At day 4 (preclinical), 11 (onset), and 28 (acute) RT-qPCR analyses of zonulin (**D**, **E**, **F**), ZO-1 (**G**, **H**,**I**), and occludin (**J**, **K**, **L**) gene expression were performed. Values expressed as means ± SEM (*n* = 14/15 per group). **(*p* < 0.01), **(*p* < 0.05), n.s.: not significant

As compared to controls, plasma zonulin levels were higher in AIA rats at the preclinical phase (+ 95%, Fig. 2A), at onset (+ 69%, Fig. 2B) but not at the acute phase (Fig. 2C). Consistent with an intestinal secretion of zonulin, ileal mRNA expression of zonulin was induced in AIA rats at the onset of arthritis (+ 360%, Fig. 2E). To clarify the mechanisms underlying the increase in intestinal permeability, ileal mRNA expression of ZO-1 and occludin, two proteins of the intestinal tight junction, were measured. Occludin and ZO-1 mRNA expressions were not different whatever the stage of arthritis (Fig. 2G to L).

### Altered intestinal epithelial integrity was observed at all stages of arthritis

**Fig. 3:**
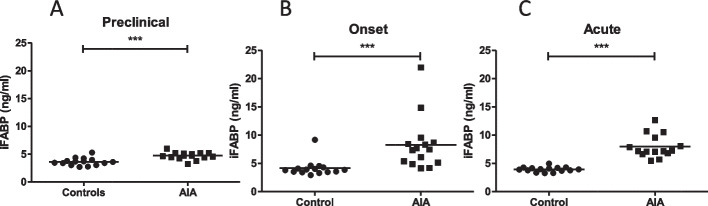
Ileal epithelial intestinal integrity in AIA rats. Plasma iFABP levels were measured in control and AIA rats at the preclinical (*A*), onset (*B*), and acute (*C*) stages. Values expressed as means ± SEM (*n* = 14/15 per group). ***(*p* < 0.0005), n.s.: not significant

As presented in Fig. 3, plasma levels of iFABP were higher in AIA rats as compared to controls at every stage of the development of arthritis.

### A transient and early intestinal dysbiosis was observed in AIA but LPS levels were not impaired

**Fig. 4:**
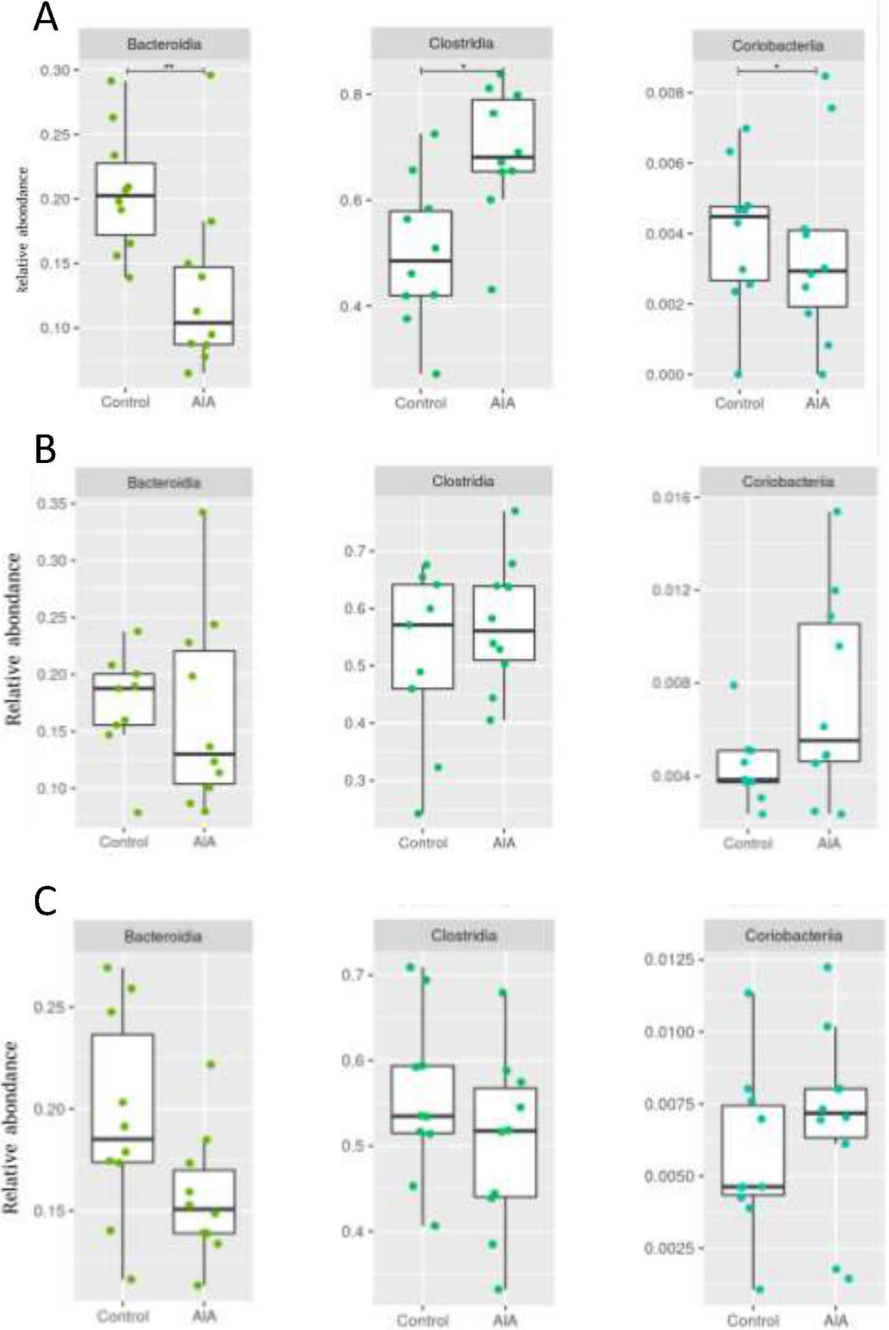
Dysbiosis in AIA rats. Relative abundance between AIA and taxonomic level controls of differentially expressed classes for samples at the preclinical (**A**), onset (**B**), and acute (**C**) stages. *n* = 10 per group. *(*p* < 0.05), **(*p* < 0.005)

**Fig. 5:**
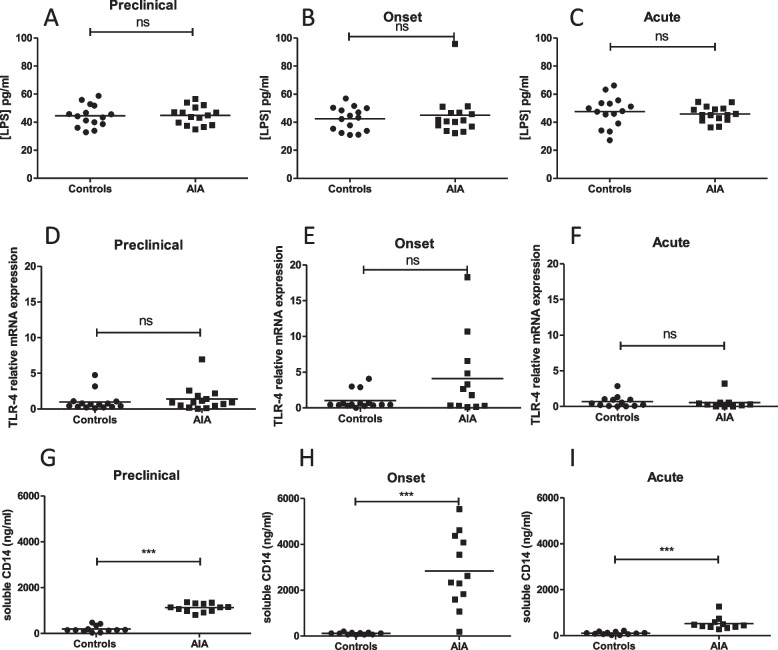
Systemic bacterial translocation in AIA rats. Plasma LPS levels were measured in control and AIA rats at the preclinical (**A**), onset (**B**), and acute (**C**) stages. At day 4 (preclinical), 11 (onset), and 28 (acute) Q-RT-PCR analyses of TLR-4 (**D**,**E**,**F**) gene expression were performed. Serum-soluble CD14 levels were measured at the preclinical (**G**), onset (**H**), and acute (**I**) stages. Values are expressed as means ± SEM (*n* = 14/15 per group). ***(*p* < 0.0001). n.s., not significant

Microbial richness and diversity were similar between control and AIA groups, regardless of the stage of development of arthritis. When we compared the microbial composition of AIA and control rats at the three stages by PERMANOVA analysis (Suppl. Figure 3), a significant difference of abundance taxa was observed at the preclinical phase (weighted UniFrac) (Fig. 4A). Analyses of the different bacterial classes in the preclinical phase showed a significantly higher proportion of *Clostridia* and a significantly lower proportion of *Bacteroidia* and *Coriobacteriia* in AIA rats (Fig. 4A). Bacterial translocation in the gut was evaluated by measuring plasma LPS levels. Regardless of the phase of arthritis, LPS levels did not differ between controls and AIA rats (Fig. 5A–C). Likewise, TLR-4 mRNA expression in the ileum of AIA was not different from controls whatever the stage of arthritis (Fig. 5D–F). Digestive bacterial translocation was also assessed at the MLN level. Genomic analysis of the MLN showed the presence of the three bacterial classes previously described in the ileum (*Bacteroidia*, *Clostridia*, *Bacilli*) but no qualitative difference was observed between the groups, regardless of the time of development of arthritis (Suppl. Figure 4). Conversely, serum sCD14 levels were greater in AIA rats compared to controls at all stages of arthritis (Fig. 5G to I).

### Correlations between arthritis, intestinal permeability, epithelial damage, inflammation, and bacterial translocation in AIA rats

Correlations are shown in Supplementary Table 2 and were searched in AIA rats from the three phases of arthritis. No correlation was found between plasma zonulin levels and arthritis and radiographic scores, LPS levels, iFABP levels, or parameters of ileal inflammation. Likewise, LPS and sCD14 levels did not correlate with arthritis and radiographic scores. Arthritis score correlated positively with iFABP levels and negatively with ileal CD8 + infiltration, IL-8, and IL-33 mRNA expression. No correlation was found between intestinal inflammation and radiographic score.

## Discussion

The major findings of the present study conducted in the rat AIA model are that (1) intestinal inflammation, transient intestinal dysbiosis, increased intestinal permeability, and intestinal barrier damage precede the onset of arthritis, (2) intestinal dysbiosis and inflammation are transient, and there is no major bacterial translocation whatever the phase of arthritis.

Zonulin, a precursor of haptoglobin 2, is a physiological modulator of paracellular permeability by controlling the organization of intercellular tight junctions, notably of certain structural junction proteins [4, 18]. In SpA patients, increased plasma zonulin levels [2] as well as increased zonulin expression in the intestine were reported as a reflection of increased intestinal permeability [2]. The present study showed that such abnormalities in zonulin plasma levels and ileum mRNA expression were also observed in the AIA model without modification of the mRNA expression of junctional proteins. The new information provided by our results is that the increase in zonulin levels occurred at the preclinical phase of arthritis and at the onset of joint symptoms but not at the acute phase of the AIA rat model. Thus, increased intestinal permeability is not the consequence of the inflammatory storm induced by joint inflammation, as confirmed by the lack of correlation between zonulin levels and arthritis or radiographical scores. Several arguments support the reversible nature of intestinal hyper-permeability. First, the fact that the mRNA expression of intestinal barrier junction proteins is not decreased and then that zonulin levels were no longer increased in AIA rats at the acute inflammatory stage. This raises the hypothesis that drugs able to reduce intestinal permeability early in the course of SpA might delay the onset of the disease. Further studies in the AIA model are warranted to check this hypothesis. Besides permeability, the present study also revealed an increase in iFABP at all stages of arthritis development, indicating alteration of intestinal epithelial integrity in agreement with increased iFABP levels previously reported in AS patients [2]. The pathophysiological role of this intestinal alteration has not been studied yet, but the loss of epithelial cells could participate in changes in intestinal permeability in early arthritis. By contrast, its persistence at the acute phase of AIA when zonulin levels were no longer increased suggests that intestinal alteration is not the main mechanism involved in gut hyper-permeability.

Intestinal inflammation is a potential culprit for increased intestinal permeability on AIA. Consistent with this, Ciccia et al. observed a significant overexpression of zonulin in the ileal samples of patients with AS, especially in those with chronic gut inflammation [2]. On the one hand, data from mice models of colitis showed that increased intestinal permeability might also induce intestinal inflammation [19]. Our results did not argue for a causality between inflammation and permeability but rather for a concomitancy of these two phenomena. Indeed, we observed that ileal mRNA expression of cytokines and lymphocyte infiltration displayed the same evolution as zonulin levels, being increased from the preclinical phase to the onset of arthritis. However, ileal inflammation but not intestinal permeability correlated with arthritis score in AIA, contrary to what is observed in the study by Audo et al. in rheumatoid arthritis [20]. Among the cytokines studied, IL-8, IL-17A, and IL-33 exhibited the earliest increase in the ileum. IL-8 is known to induce transendothelial migration of neutrophils to sites of inflammation, including joints [21], and circulating levels of IL-8 are strongly correlated with disease and inflammatory activities in AS patients [22]. Our data showing that IL-17 upregulation preceded that of IL-23 and TNFα support the fact that IL-23 and IL-17 are at least partly uncoupled in SpA and suggest that IL-17 plays an early role in the pathophysiology of the disease. It is consistent with the superior efficacy of IL-17 inhibitors in TNF-naive patients than in TNF-experienced patients [23, 24]. Moreover, previous data reported that after acute intestinal injury, IL-17 played a role in the protection of epithelial barriers [25, 26] but in an inflammatory environment, IL-17 could lead to inflammatory cascades [27]. Likewise, IL-33 is a cytokine whose role appears to be context-dependent. IL-33 behaves as an alarmin in response to epithelial cell injury by triggering an innate immune response, but could also induce a Th2 type response in order to maintain tissue integrity [28]. In SpA, circulating levels of IL-33 were increased [29] and in IBD, tissue expression of IL-33 was enhanced and increased in cases of intestinal inflammation [28]. Further studies are needed to better characterize the role of IL-33 in intestinal permeability but a previous study revealed that the development of antigen-induced arthritis or CIA was not impaired in IL-33 deficient mice [30], suggesting that IL-33 is more a regulating than a pivotal cytokine in arthritis development. Evaluation of other cytokines such as IL-1 or IFN-_γ_ would be of interest to refine our data and requires further studies.

An important result of our study is that alterations of gut permeability and inflammation occurred in the presence of a very early and transient dysbiosis in the rat AIA model. This provides three important information: (i) intestinal alterations occur before joint involvement, (ii) dysbiosis occurs in parallel to intestinal permeability and inflammation, and (iii) dysbiosis is not necessary for the maintenance of the disease in the AIA rat model. These data indicated that dysbiosis could be required to induce changes in the intestinal barrier and could modulate the onset of arthritis. This was suggested by the dysbiosis in animal models of SpA such as the B27 transgenic rat model, having intestinal inflammation but in which arthritis is not systematically present [31], and in patients with SpA [32]. Is intestinal dysbiosis the trigger for the observed intestinal alterations? This attractive hypothesis needs to be tested. Indeed, a recent publication in an animal model of rheumatoid arthritis showed that dysbiosis alone was not responsible for the increase in intestinal permeability and that dysbiosis did not occur in the early stage of the disease [33]. The study by Guggino et al. showed that intestinal dysbiosis activates the inflammasome that modulates the production of IL-23 and that LPS could be responsible for the inflammasome up-regulation in peripheral blood mononuclear cells of AS patients [34]. However, our study did not reveal any increased LPS level nor any lymphatic translocation suggesting that bacterial translocation is not a *sine qua non* condition for the onset of intestinal permeability or joint involvement in the AIA model. In our study, bacterial translocation was evaluated through the measurement of circulating LPS levels, sCD14, and ileal mRNA expression of TLR-4. While LPS levels and mRNA expression of TLR-4 were unchanged in AIA rats compared to controls, sCD14 levels were increased at all phases of arthritis, but they did not correlate with zonulin levels in AIA rats, contrary to what was observed in patients with rheumatoid arthritis [35]. As sCD14 enhances the response of TLR-4-expressing cells by facilitating the binding of LPS to its receptor [36, 37], one hypothesis is that sCD14 is not a relevant marker of bacterial translocation but rather reflects the systemic inflammation in this model, as previously described [38].

The present study was conducted in the AIA model in rats. This model is widely used as a model of rheumatoid arthritis, but also exhibits features of reactive arthritis. Of course, a difference between the clinical situation in which a preceding infection leads to arthritis is that here the injected Mycobacteria are dead. However, although the bacteria are dead, the principle of antigenic peptide presentation remains the essential element of this model together with the production of inflammatory factors. Moreover, this model is characterized by an ankylosis of the tail, comparable to the spinal ankylosis in patients with SpA [39] as well as with a great sensibility to non-steroidal anti-inflammatory drugs as in SpA [40]. In this model, the role of Mycobacteria alone in the intestinal changes is questionable. Indeed, in a study in a mouse model of uveitis induced by killed *Mycobacterium tuberculosis* associated or not with interphotoreceptor to retinoid-binding protein, Mycobacterium alone was able to induce a significant dysbiosis. However, in this latter study, Mycobacterium alone was not sufficient to enhance intestinal permeability or to alter intestinal T cell subsets [41]. The present study included only male rats, because male rats are known to be more susceptible to the AIA model, with a 100% incidence of arthritis [14, 42]. In addition, this study was made in the following of our previous study showing an early endothelial dysfunction in mesenteric arteries in male Lewis rats [11]. Whether different results would have been obtained using female rats requires further investigation.

Our study has several limitations, first of all, the use of indirect methods for the evaluation of intestinal permeability, bacterial translocation, and intestinal integrity. Regarding intestinal permeability, it would be interesting to complete our data with the FITC-Dextran method. In view of the discrepancy between the increase in intestinal permeability and the absence of changes in the mRNA expression of occludin and ZO-1, the measure of expression of occludin and ZO-1 at the protein level, along with other junctional proteins would have been relevant. In order to further evaluate the hypothesis of hyperpermeability secondary to an alteration of the intestinal barrier, other markers of the integrity of the intestinal epithelial barrier could have been evaluated, like paneth cell degranulation or goblet cell loss. Regarding intestinal inflammation, it would have been interesting to study precisely the leukocytes populations at the intestinal level to better understand the different cytokine expression changes. We can indeed wonder about the production of IL-4 and IL-17 derived from infiltrating T cells. A more precise study of Th17 cells could have clarified their anti- or pro-inflammatory feature and potentially their involvement in the transient nature of dysbiosis. Finally, a study of gram-negative bacteria could be done to understand the absence of variation in LPS, a marker of gram-positive bacteria. Finally, analyses were carried out only on the ileal and not the colonic segment. These limitations allow us to envisage further studies to confirm and clarify our results. The use of drugs allowing the restoration of intestinal tight junctions will be required to confirm the importance of intestinal damage in the occurrence of arthritis in the AIA model.

## Conclusion

In conclusion, the present study shows that intestinal inflammation and alterations of the intestinal barrier are not the consequence of joint damage but precede arthritis development in the AIA rat model. The fact that intestinal changes occurred in the absence of bacterial translocation did not support a key role of the systemic passage of intestinal bacterial antigens in intestinal inflammation and permeability in arthritis. The reversible nature of the intestinal alteration offers interesting therapeutic perspectives targeting the gut to hamper the development of SpA. Overall, these data show that intestinal changes precede the development of arthritis but argue against a strict “correlative” model in which arthritis and gut changes are inseparable.

## Supplementary Information

**Additional file 1: Supplementary Table 1.** Primer sequences used in RT-qPCR. **Supplementary Table 2.** Correlation analysis in AIA rats at different stages of arthritis. **Suppl. Fig. 1.** Radiographs of hind paws. **Suppl. Fig. 2.** Composition of intestinal microbiota in AIA rats compared to control rats at days 4, 11, and 28. **Suppl. Fig. 3.** Microbiota of mesenteric lymph nodes in AIA rats compared to control rats.

## Data Availability

The data underlying this article will be shared on reasonable request to the corresponding author.

## Abbreviations

AIA: Adjuvant-induced arthritis
ARRIVE: Animal Research: Reporting of In Vivo Experiments
BT: Bacterial translocation
iFABP: Intestinal fatty acid binding protein
I-Inf: Intestinal inflammation
IP: Intestinal permeability
LCMS2: Liquid chromatography coupled mass spectrometry
MLN: Mesenteric lymph nodes
PERMANOVA: Parametric multivariate analysis of variance
RT-qPCR: Real-time quantitative
sCD14: Soluble CD14
SpA: Spondyloarthritis
