# Supplementary material for: Increased gut permeability and intestinal inflammation precede arthritis onset in the adjuvant-induced model of arthritis
Source: Arthritis Res Ther. 2023 Jun 6;25:95. doi: 10.1186/s13075-023-03069-9 (PMC10242991; doi:10.1186/s13075-023-03069-9)
Supplement: Supplementary file 1 — Additional file 1: Supplementary Table 1. Primer sequences used in RT-qPCR. Supplementary Table 2. Correlation analysis in AIA rats at different stages of arthritis. Suppl. Fig. 1. Radiographs of hind paws. Suppl. Fig. 2. Composition of intestinal microbiota in AIA rats compared to control rats at days 4, 11, and 28. Suppl. Fig. 3. Microbiota of mesenteric lymph nodes in AIA rats compared to control rats. [file 13075_2023_3069_MOESM1_ESM.docx]

**Supplemental data**

**Increased gut permeability and intestinal inflammation precede arthritis onset**

**in the adjuvant-induced model of arthritis**

*Sophie Hecquet (MD)^1,2^, Perle Totoson (PharmD, PhD)^1^, Hélène Martin (PhD)^1^,*

*Marie-Paule Algros (MD) ^3^, Philippe Saas (PharmD, PhD) ^4,5^, Jean-Paul Pais-de-Barros (PhD) ^5,6^, Alban Atchon (MSc)^7^, Benoît Valot (PhD)^7^, Didier Hocquet (PharmD, PhD) ^7,8^, Maude Tournier ^1^,*

*Clément Prati (MD, PhD) ^1,2^, Daniel Wendling (MD, PhD)^2,9^, Céline Demougeot (PharmD, PhD)^1 *^, Frank Verhoeven (MD, PhD)^1, 2*^*

* contributed equally to the manuscript

^1^ Université de Franche-Comté, PEPITE, F-25000 Besançon, France

^2^ Service de Rhumatologie, CHRU Besançon, F-25000 Besançon, France

^3^ Service d’anatomopathologie, CHRU Besançon, F-25000 Besançon, France

^4^ Université de Franche-Comté, INSERM, EFS BFC, UMR1098 RIGHT, F-25000 Besançon, France

^5^ Lipidomic Analytic Platform LabEX LipSTIC, INSERM, LNC UMR1231, F-21000, Dijon, France.

^6^ Université de Bourgogne, INSERM UMR1231, F-21000 Dijon, France.

^7^ Université de Franche-Comté, Bioinformatique et Big Data au Service de la Santé, UFR Sciences de la Santé, F-25000 Besançon, France

^8^ Université de Franche-Comté, UMR CNRS 6249 Chrono-environnement, F-25030 Besançon, France

^9^ Université de Franche-Comté, EPILAB EA 4266 Pathogènes et Inflammation, F-25000 Besançon, France

**^#^ Corresponding author:**

Prof. Céline DEMOUGEOT

PEPITE EA 4267, UFR Sciences de Santé, 19 rue Ambroise Paré, bâtiment S, 25000 Besançon, France.

Tel: (33) 3 63 08 23 25

E-mail: [cdemouge@univ-fcomte.fr](mailto:cdemouge@univ-fcomte.fr)

The authors declare no conflict of interest in relation to this work.

**Supplemental methods**

*Induction, clinical follow-up and evaluation of arthritis*

Arthritis was induced in rats by a single intradermal injection at the base of the tail of 120 μl of 1 mg of heat killed *Mycobacterium butyricum* (Difco, Detroit, USA) suspended in 0.1 ml of Freund's incomplete adjuvant (Difco). Non-arthritic age-matched rats were used as controls and received the same volume of saline (120 µL) using the same procedure. The adjuvant-induced-arthritis (AIA) model is characterized by rapid onset and progression of a measurable reactive polyarthritis. Arthritis is clinically characterized by severe erythema and diffuse soft tissue swelling with paw malformations reducing locomotor activity and is frequently associated with tail inflammation, ankylosis of the base of the tail, weight loss, anorexia, and diarrhea [1]. Rats were weighed daily and examined 5 days per week for clinical signs of arthritis and to assess any signs of animal suffering. An arthritis score was assigned to each rat as follows [2]: 0.1= arthritis of 1 digit, 0.5= weak and moderate arthritis of 1 big joint (ankle or wrist), and 1= intense arthritis of 1 big joint. The tarsus and the ankle were considered the same joint. The sum of the joint scores in the 4 limbs gives a maximum arthritis score of 6 for each rat. Ankle diameter was measured with digital calipers (Vernier Stainless). As arthritis models are models inducing severe pain, the animal's well-being was evaluated from day 10 post-induction (onset of symptoms) according to Lloyd and Wolfensohn's score [3]. The parameters evaluated were the appearance of the rat (0-4), its natural behaviour (0-3), its state of hydration (0; 5), its respiration (0-4) and its induced behaviour (0-3).

*Blood analysis*

Intestinal permeability was evaluated by measuring plasma level of zonulin using ELISA kit (MyBiosource, San Diego, USA). Epithelial integrity of intestinal barrier was assessed by measuring plasma level of intestinal fatty acid binding protein (iFABP) using ELISA kit (Abbexa, Coger). Bacterial translocation was assessed by measuring plasma level of LPS by liquid chromatography coupled mass spectrometry (LCMS2) and serum level of CD14 soluble (sCD14), which facilitates the binding of LPS to its TLR-4 receptor [4], by ELISA test (Cusabio, Antibodies online). Limits of detection for zonulin, iFABP, LPS and sCD14 were 0.5 ng/mL, 0.1 ng/mL, 18 ng/mL, 7 ng/mL, respectively.

*Immunohistology of ileum*

First, 4-μm-thick tissue sections were cut from paraffin-embedded ileum and placed on positively charged slides. Immunostaining was performed on the Ventana Benchmark ULTRA automated slide stainer (Ventana Medical Systems, Tucson, AZ, USA). Sections were dewaxed in xylene and rehydrated using a graded series of alcohol. Antigen retrieval was carried out by incubating slides for 60 minutes in CC1 buffer. Sections were then incubated for 32 minutes with the primary antibodies targeting CD3, CD4 and CD8 (Agilent, Leica, Diagomics respectively). The staining was visualized using the Ventana Ultraview DAB Detection System. For quantification of immune cells, sections were digitally scanned (Nanozoomer 2.0, Hamamatsu) and imported into QuPath 0.2.3 software The evaluation of the number of positive cells per mm² has been done by computerized counts on the manually contoured epithelial areas.

*Quantitative Real Time Polymerase Chain Reaction*

Expression of mRNAs of different targets was measured in ileums by rt-qPCR. Frozen ileums were grinded in a mortar, mRNAs were extracted using Qiagen^®^ mRNA extraction kit, then rt-qPCR were performed using Biorad^®^ cDNA and SybrGreen kits. mRNAs expression of IL-23 (p19 subunit), IL-17A, IL-33 and CXCL1 (murine equivalent of IL-8) was measured to assess intestinal inflammation, zonulin, ZO-1 and occludin to assess integrity of barrier function, and Toll-like-Receptor 4 (TLR-4) to determine bacterial translocation.

All the samples were deposited in duplicates. Two negative controls were used on each plate: a RNAse-free water and a no-RT samples. The thermocycler conditions were 3 min at 95°C to allow polymerase activation then 40 PCR cycles at 95°C for 15 s and at 60°C for 60 s. Normalized and averaged fluorescence ratios of targets were used to calculate the fold changes in samples from the different rat groups.

*Metagenomic analysis*

A sequencing of the V3-V4 region of 16S rRNA was performed on Illumina MiSeq (v2) in pair-end (2x250 bp) for 60 fecal samples and 60 Mesenteric lymph nodes (MLN) (10 per each group). On our sequencing data set (fastq) we run DADA2 [5], a software that designs and corrects the amplicon errors sequenced by Illumina. The quality control of the reads, the correction of the amplicon errors and the construction of the amplicon sequence variants (ASV) table [6] have been performed with DADA2. The identified ASVs were assigned taxonomy via the IDTAXA method [7] implemented in the DECIPHER package (https:// bioconductor.org/packages/release/bioc/html/DECIPHER.html), with the SILVA database (https://www.arb-silva.de/). The Phyloseq package [8] was used for importing, storing, analyzing and plotting the data grouped into ASVs. Comparisons of alpha and beta diversity were performed according to study groups (AIA-Control and/or Day 4, Day 11 and Day 28). Principal coordinate analysis (PCoA) was performed on the UniFrac [9] distance to illustrate microbial communities according to rat groups. A non-parametric multivariate analysis of variance (PERMANOVA) approach was used to compare dissimilarities between bacterial communities. We performed a compositional analysis(11) of the microbiota to distinguish differentially abundant taxa between AIAs and controls.

**Supplemental tables**

**Supplementary Table 1. Primer sequences used in RT-qPCR.**

| Target | Forward primer (5’→ 3’) | Reverse primer (5’→3’) |
| --- | --- | --- |
| CXCL1 (IL8) | CCAGCCACACTCCAACAGAGCA | GGCGCCCCTGTGGCTTGG |
| IL17A | AACAGAGACCTGAGGCTA | TCCATATCACTTGCTGAGATT |
| IL23p19 | ACACACACCAGTGGGACAAAT | TCCTTTGCAAACAGAACTGGCT |
| IL33 | CTGGCACTTACATAGGAG | CAGTTGGAAGCATTGAAC |
| TNF-α | CCA-ATC-TGT-GTC-CTT-CTA-A | TTC-TGA-GCA-TCG-TAG-TTG |
| Zonulin | ACT-GGG-TCC-AGG-AAA-CAA-TG | TCC-TCT-TCC-AGG-GTG-AAT-TG |
| Occludin | TGA-GCC-CGA-GTG-GAA-AGG-AC | GCA-TGA-AGG-ACT-TCC-CAG-AGT |
| ZO-1 | ATG-GTT-GGT-ATG-GTG-CCC-TG | TTG-TAG-CAC-CAT-CCG-CCT-TC |
| TLR-4 | ATC-TGA-GCT-TCA-ACC-CCC-TG | TGT-CTC-AAT-TTC-ACA-CCT-GGA-T |
| β-actine | TATCGGCAATGACGCGTTCC | TGCCTGGGTACATGGTGGTG |
| GAPDH | GGGCATCCTGGGCTACACTG | GAGGTCCACCACCCTGTTGC |

**Supplementary Table 2. Correlation analysis in AIA rats at different stages of arthritis.**

|  | r | p | n |
| --- | --- | --- | --- |
| Body weight & [zonulin] | 0.179 | 0.249 | 43 |
| [zonulin] & [LPS] | 0.155 | 0.319 | 43 |
| Arthritis score & [zonuline] | -0.099 | 0.608 | 29 |
| Arthritis score & [LPS] | 0.024 | 0.899 | 30 |
| **Arthritis score & radiographic score** | **0.93** | **<0.0001** | **43** |
| Radiographic score & [zonulin] | -0.01 | 0.946 | 43 |
| Radiographic score & [LPS] | 0.003 | 0.984 | 43 |
| **Arthritis score & IL-8 ileal expression** | **-0.33** | **0.03** | **42** |
| Arthritis score & IL-33 ileal expression  Arthritis score & IL-17 ileal expression  **Arthritis score & IL-23 ileal expression**  Arthritis score & TNF-α ileal expression  Arthritis score & CD4 lymphocyte  Arthritis score & CD8 lymphocyte  **Arthritis score & iFABP**  [Zonulin] & iFABP  [Zonulin] & IL-8 ileal expression  [Zonulin] & IL-33 ileal expression  [Zonulin] & IL-17 ileal expression  [Zonulin] & IL-23 ileal expression  [Zonulin] & TNF-α ileal expression  [Zonulin] & Zonulin ileal expression  [Zonulin] & [sCD14]  Arthritis score & [sCD14] | -0.13  -0.3  **-0.59**  -015  0.4  0.26  **0.7**  0.14  -0.69  0.12  -0.007  0.04  0.05  0.08  0.25  -0.31 | 0.42  0.05  **<0.0001**  0.34  0.02  0.88  **<0.0001**  0.38  0.66  0.46  0.96  0.78  0.75  0.61  1.14  0.07 | 40  42  **42**  32  30  30  **43**  43  43  41  43  43 43  40  34  33 |

LPS=lipopolysaccharide, iFABP=intestinal fatty acid binding protein, sCD14=soluble CD14

**Supplemental figures**

**
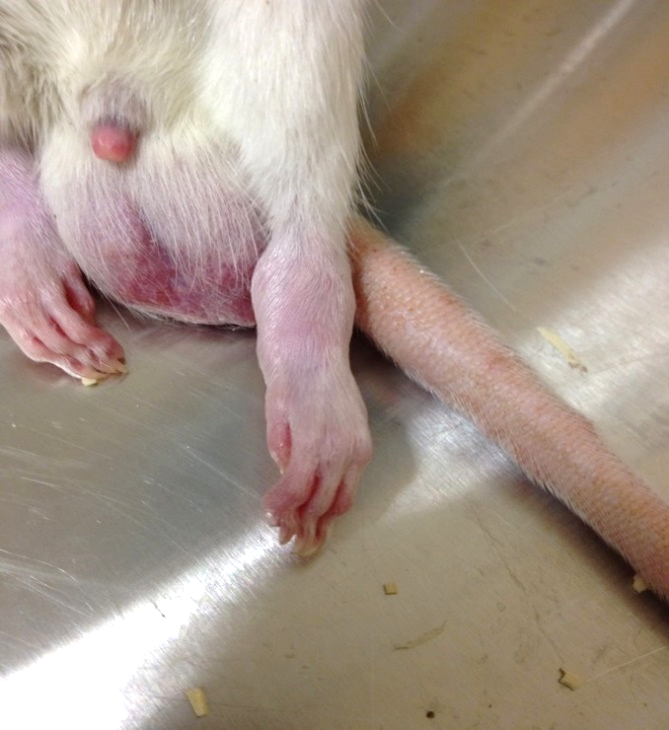
**

**A**

**B C**

**
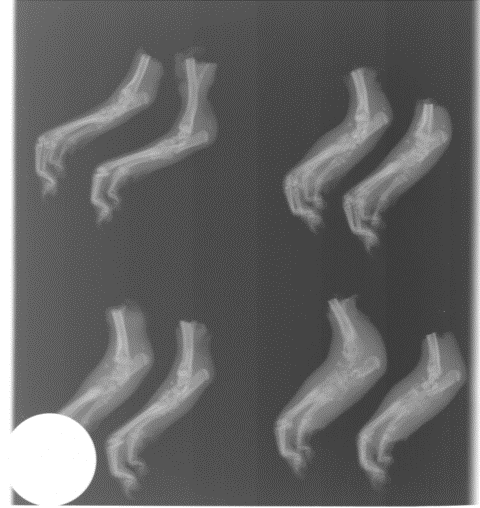

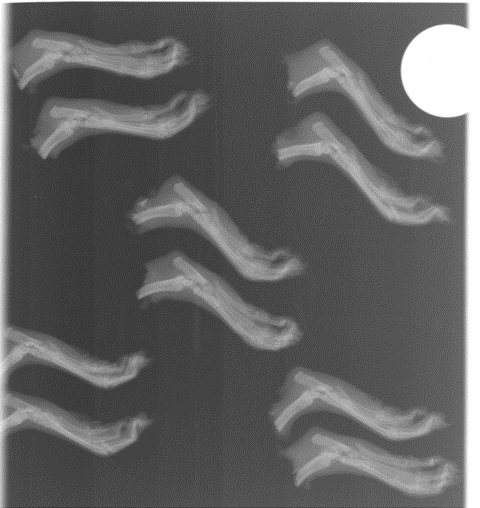
**

**Suppl. Fig. 1. Radiographs of hind paws.** Example of arthritis in an AIA rat (A). Example of radiographs of normal hind paws (control rat, B) and hind paws in AIA rats with soft tissue swelling, demineralization, loss of cartilage, bone erosions and bone formation (C).

**
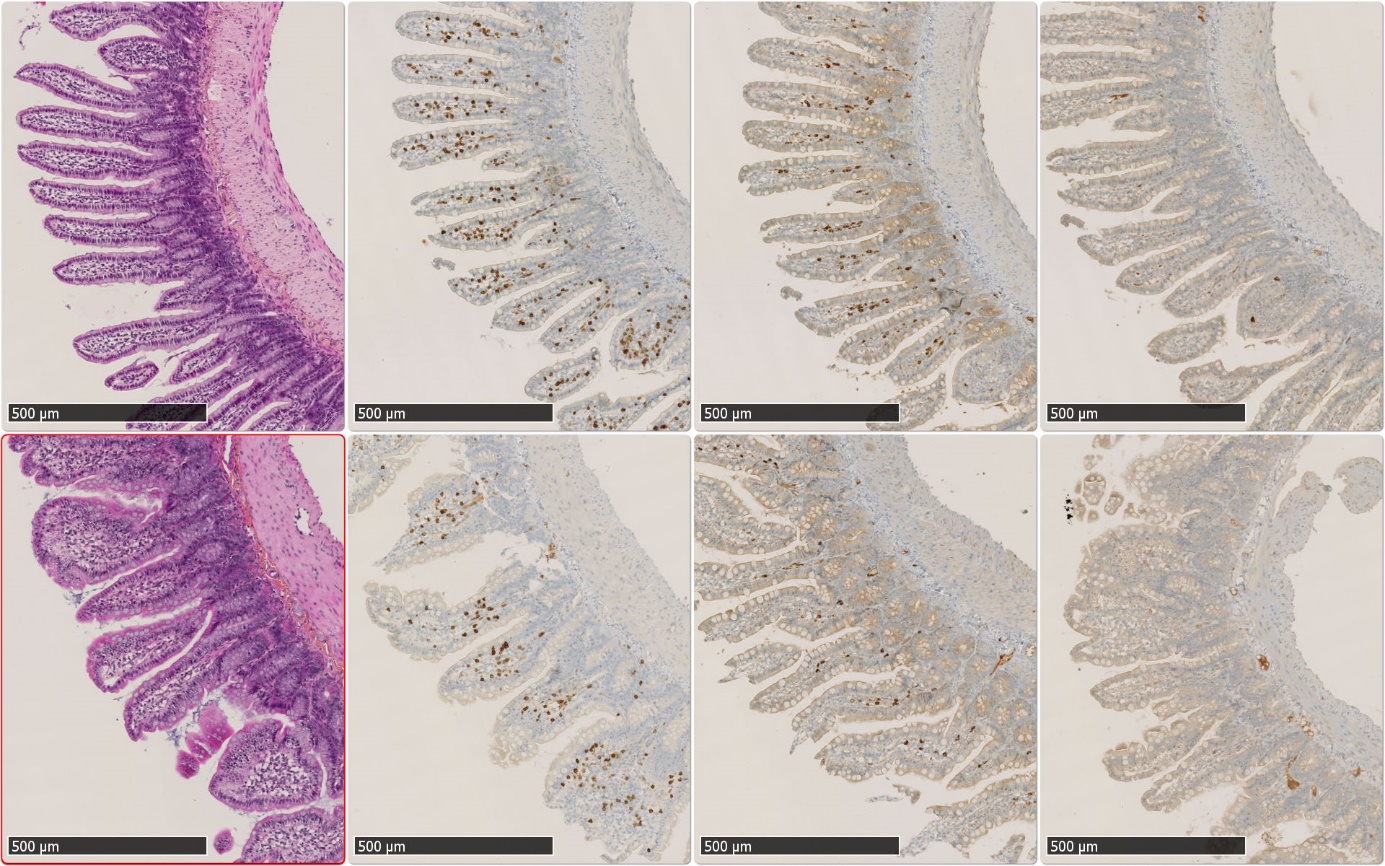
**

B D F

A C E

**Suppl. Fig. 2. Immunohistology of ileum**. Ileal section of AIA and control rats at day 4. Number of CD3^+^, CD4^+^ and CD8^+^ T lymphocytes in AIA (A, C, E) and controls (B, D, F) rats respectively.


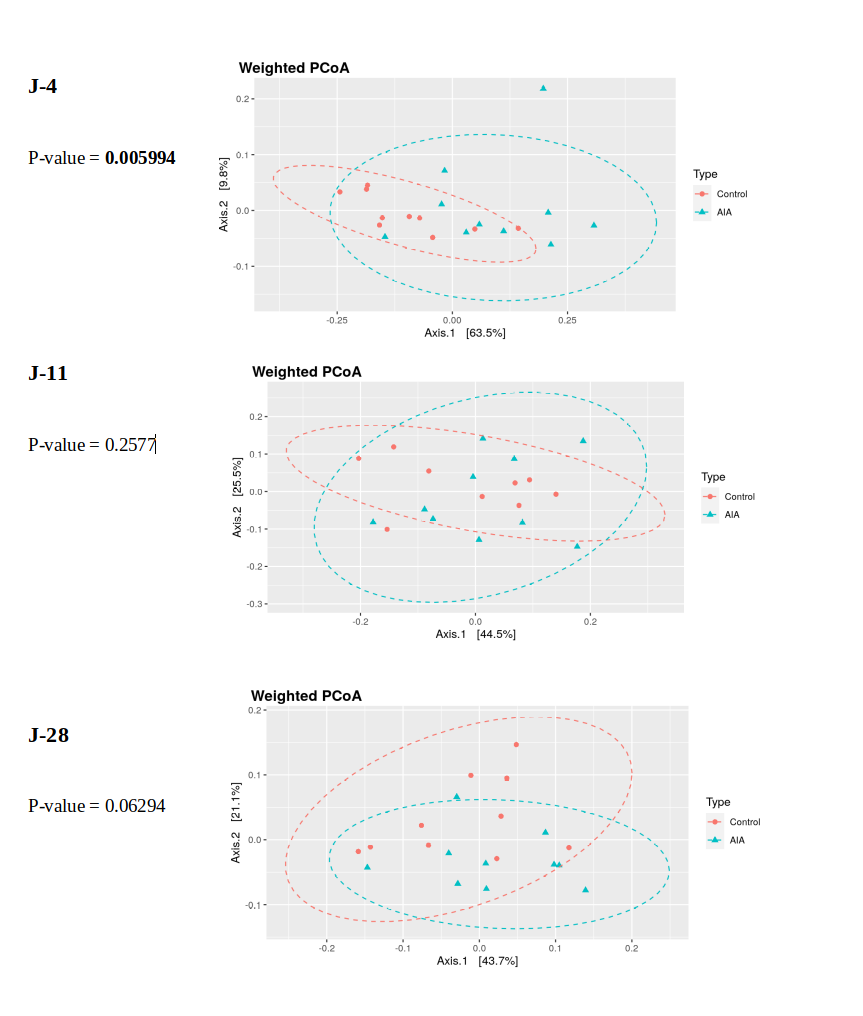


**Day 28**

**Day 11**

**Day 4**

**Suppl. Fig. 3. Composition of intestinal microbiota in AIA rats compared to control rats at days 4, 11, and 28.** Comparison of Principal coordinate analysis (PCoA) of gut microbiota samples from rats control and AIA rats between the days of sampling. Figures are obtained from weighted UniFrac (left panels) distances. Weighted PcoA captures the relative abundance of taxa shared between samples. P-values were obtained by PERMANOVA and were significant if ≤ 0.05.


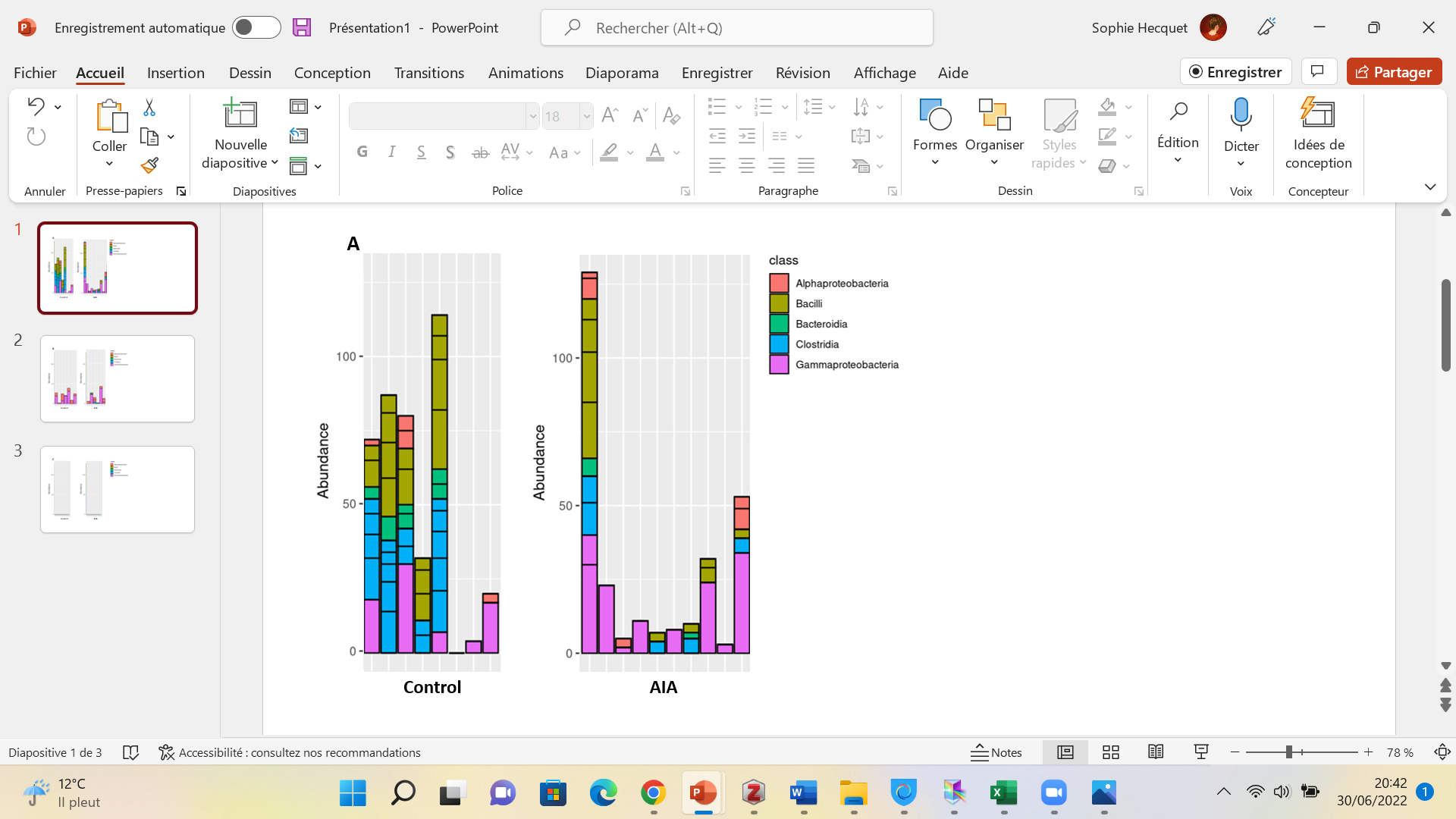

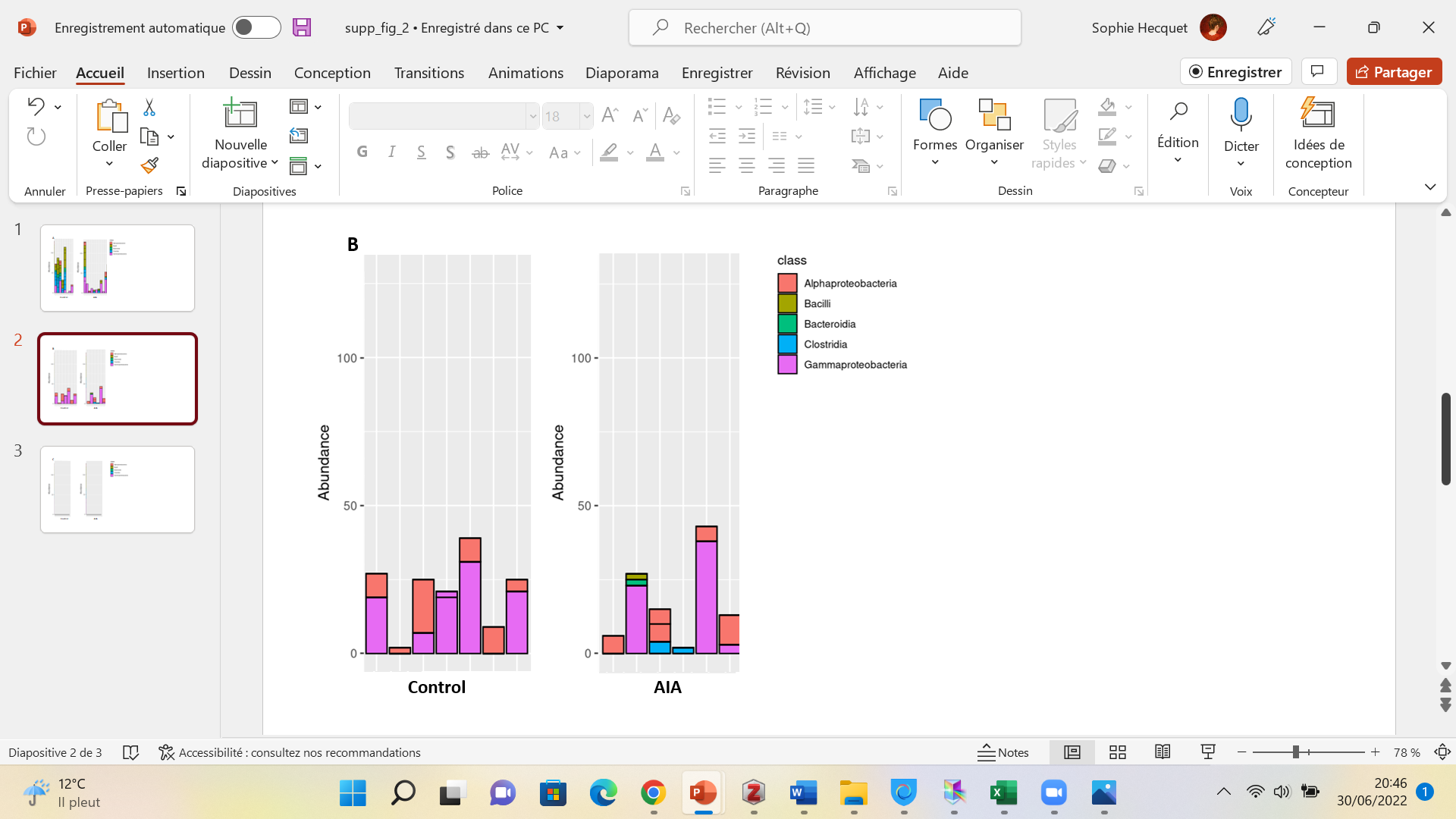


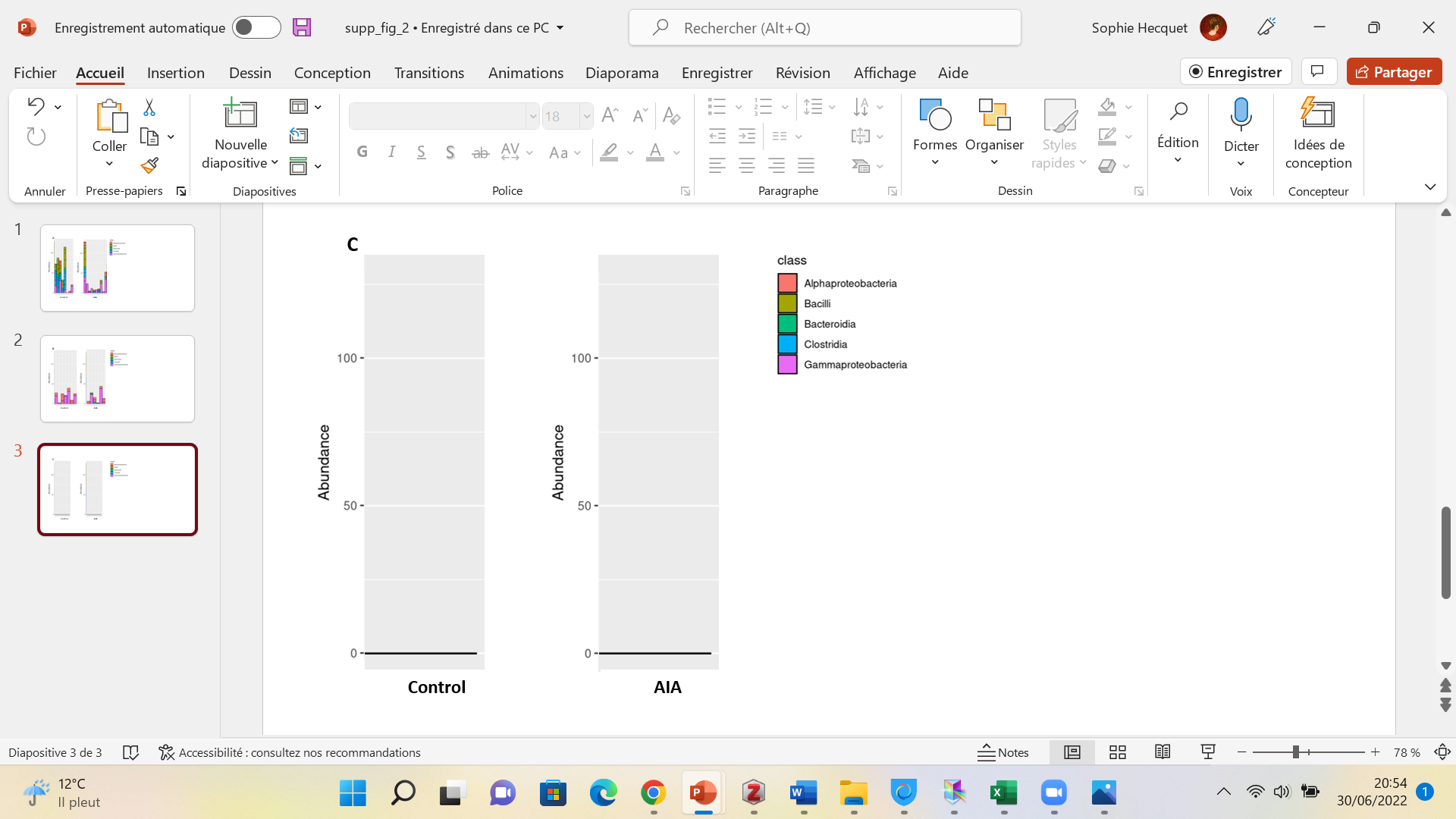


**Suppl. Fig. 4. Microbiota of mesenteric lymph nodes in AIA rats compared to control rats.**

Abundance of amplicon sequence variants in AIA and control rats distributed by bacterial classes at the preclinical (A), onset (B), and acute (C) stages. n= 10 per group (samples are not shown if no class is identified)"

.

**Supplemental References**

1 Oliver SJ, Brahn E. Combination therapy in rheumatoid arthritis: the animal model perspective. *J Rheumatol Suppl* 1996;**44**:56–60.

2 Sakaguchi N, Takahashi T, Hata H, *et al.* Altered thymic T-cell selection due to a mutation of the ZAP-70 gene causes autoimmune arthritis in mice. *Nature* 2003;**426**:454. doi:10.1038/nature02119

3 Wolfensohn SE, Lloyd M. Practical use of distress scoring systems in the application of humane end points. In: Hendriksen C, Morton D, eds. *Humane endpoints in animal experiments for biomedical research*. Royal Society of Medicine Press 1999. https://epubs.surrey.ac.uk/827030/ (accessed 27 Oct 2020).

4 Luchetti MM, Ciccia F, Avellini C, *et al.* Gut epithelial impairment, microbial translocation and immune system activation in inflammatory bowel disease-associated spondyloarthritis. *Rheumatology (Oxford)* Published Online First: 23 May 2020. doi:10.1093/rheumatology/keaa164

5 Callahan BJ, McMurdie PJ, Rosen MJ, *et al.* DADA2: High-resolution sample inference from Illumina amplicon data. *Nat Methods* 2016;**13**:581–3. doi:10.1038/nmeth.3869

6 Callahan BJ, McMurdie PJ, Holmes SP. Exact sequence variants should replace operational taxonomic units in marker-gene data analysis. *ISME J* 2017;**11**:2639–43. doi:10.1038/ismej.2017.119

7 Murali A, Bhargava A, Wright ES. IDTAXA: a novel approach for accurate taxonomic classification of microbiome sequences. *Microbiome* 2018;**6**:140. doi:10.1186/s40168-018-0521-5

8 McMurdie PJ, Holmes S. phyloseq: an R package for reproducible interactive analysis and graphics of microbiome census data. *PLoS One* 2013;**8**:e61217. doi:10.1371/journal.pone.0061217

9 Lozupone C, Lladser ME, Knights D, *et al.* UniFrac: an effective distance metric for microbial community comparison. *ISME J* 2011;**5**:169–72. doi:10.1038/ismej.2010.133
